# Performance Analysis of Global Navigation Satellite System Signal Acquisition Aided by Different Grade Inertial Navigation System under Highly Dynamic Conditions

**DOI:** 10.3390/s17050980

**Published:** 2017-04-28

**Authors:** Chunxi Zhang, Xianmu Li, Shuang Gao, Tie Lin, Lu Wang

**Affiliations:** School of Instrument Science and Opto-electronics Engineering, Beihang University, Beijing 100191, China; zhangchunxi@buaa.edu.cn (C.Z.); gaoshuang@buaa.edu.cn (S.G.); lintie@buaa.edu.cn (T.L.); wanglu@buaa.edu.cn (L.W.)

**Keywords:** INS-aided acquisition, Doppler shift estimation error, code phase estimation error, high dynamic, GNSS signal

## Abstract

Under the high dynamic conditions, Global Navigation Satellite System (GNSS) signals produce great Doppler frequency shifts, which hinders the fast acquisition of signals. Inertial Navigation System (INS)-aided acquisition can improve the acquisition performance, whereas the accuracy of Doppler shift and code phase estimation are mainly determined by the INS precision. The relation between the INS accuracy and Doppler shift estimation error has been derived, while the relation between the INS accuracy and code phase estimation error has not been deduced. In this paper, in order to theoretically analyze the effects of INS errors on the performance of Doppler shift and code phase estimations, the connections between them are re-deduced. Moreover, the curves of the corresponding relations are given for the first time. Then, in order to have a better verification of the INS-aided acquisition, a high dynamic scenario is designed. Furthermore, by using the deduced mathematical relation, the effects of different grade INS on the GNSS (including Global Positioning System (GPS) and BeiDou Navigation Satellite System (BDS)) signal acquisition are analyzed. Experimental results demonstrate that the INS-aided acquisition can reduce the search range of local frequency and code phase, and achieve fast acquisition. According to the experimental results, a suitable INS can be chosen for the deeply coupled integration.

## 1. Introduction

The Global Navigation Satellite System (GNSS), using space satellites to achieve positioning and navigation, is widely used in civil and military applications, such as positioning, timing, and navigation. At present, the main GNSS in the world include the Global Positioning System (GPS) of the United States, the Global Navigation Satellites System (GLONASS) of Russia, the BeiDou Navigation Satellite System (BDS) of China and the Galileo Navigation Satellite System (Galileo) of the European Union [1,2]. However, their performance may be subject to the impact of environmental factors, including signal interference and dynamic factors. The Inertial Navigation System (INS) can provide continuous high-precision position, velocity and attitude data for a short time, but after a while gyro and accelerometer errors accumulate and the navigation errors grow. The integration of GNSS/INS has many peculiarities and offers a way for high-accurate positioning. There are three architectures of integrated navigation systems: loosely coupled, tightly coupled and deeply coupled (also called ultra-tightly coupled) [3,4,5]. The deeply coupled integration has been widely studied in recent years, due to the fact that it can achieve the fusion of GNSS and INS information at the signal processing level. What’s more, it has better performances in highly dynamic environments due to the adaptable dynamic characteristics of INS. However, most research about GNSS/INS integration is concentrated on integration architectures and solutions instead of the signal acquisition issues. 

Acquisition is the first step of the signal processing for a GNSS receiver, and it is a time-consuming process. Under highly dynamic conditions, the Doppler shift between the carrier and the satellite changes greatly, resulting in a larger frequency search range and longer search time. According to the nature of signal acquisition, the common approach for improving signal acquisition performance is by reducing the carrier frequency and code phase search range. Compared with unaided acquisition methods, the INS-aided acquisition has many advantages under highly dynamic and harsh environment conditions, such as the lower C/N_0_ (carrier to noise ratio) signal acquisition for the higher sensitivity receivers, and the accurate estimation of Doppler frequency shift and code phase to reduce the search space [6,7]. Because of these outstanding performance features, it has been studied for many different applications [7,8,9,10,11].

Progri and Alban made the effort to investigate the methodology of the Doppler estimation [12,13]. In [14], the effects of IMU accuracy on the Doppler estimation error were analyzed. Ye and He presented the INS-aided acquisition scheme and analyzed the acquisition performance [15,16]. Reference [17] presented a fast acquisition method for GPS receivers aided by INS information and performed acquisition experiment under low dynamics. However, the addressed methods have some disadvantages: (i) the traditional INS-aided acquisition mostly uses INS velocity information to estimate the carrier Doppler frequency shift without reducing the uncertainty of code phase search; (ii) the mathematical model of Doppler shift and code phase estimation errors contributed by the INS velocity error and position error were not derived in detail; (iii) the literature has been focused on low dynamics, and not on high dynamics situations. Hence, due to the small Doppler shift caused by low dynamics, the former studies have had limited success in proving any INS-aided acquisition performance improvement. Furthermore, concerning the limitations under high dynamics, there is a little related literature. Thus, the performance analysis of GNSS signal acquisition aided by different grade INS under high dynamic conditions is still necessary.

**Figure 1:**
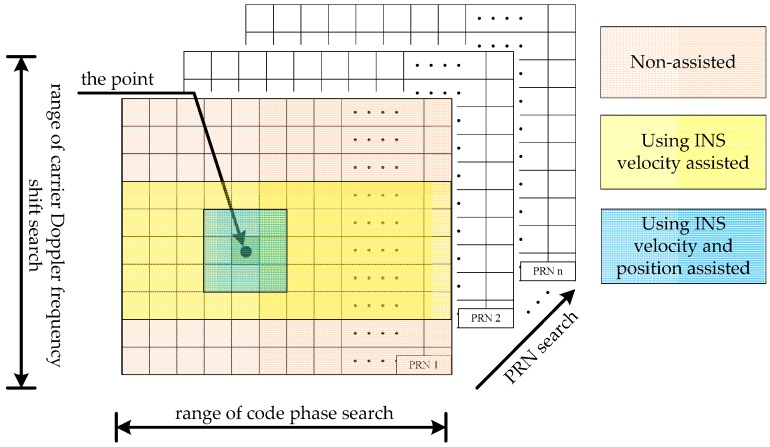
Schematic diagram of the search scope under three conditions.

Figure 1 illustrates in a schematic diagram the search scope under three conditions: non-assisted acquisition, INS velocity-assisted acquisition and INS velocity, position-assisted acquisition. Both the range of carrier Doppler frequency and code phase are reduced by using INS velocity- and position-assisted acquisition.

The Doppler frequency shift is related to the relative position and velocity of the receiver with respect to the transmitter. The former research mainly concentrated in low dynamic environments, such as vehicles and handheld devices. The velocity and position changes are small under such conditions, so the Doppler frequency shift is relatively small. However, in a high dynamics environment, such as missiles and aircrafts, the Doppler frequency shift is large. Therefore, this paper defines a high dynamic scene which includes acceleration, uniform motion, turning and climbing. In this high dynamic scene, the GNSS acquisition performance aided by different grade INS can be fully verified.

In the INS-aided acquisition, the INS accuracy has crucial effects on the Doppler and the code phase estimation errors. Different grade INS devices afford different velocity errors and position errors. In order to analyze the influence of different INS grades on acquisition performance, the mathematical model between them is deduced in detail. Furthermore, the relationships between INS accuracy, Doppler estimation error, code phase error and acquisition performance are verified by signal acquisition experiments which use the trajectory of the above high dynamic scene. Finally, the influences of different grades of INS on the acquisition performance and the tracking performance are analyzed.

BDS is the independently developed Chinese global navigation satellite system, which has many similarities with GPS. Based on the analysis of the characteristics of GPS L1 frequency and BDS B1I signals modulation, the INS-aided acquisition performance is proved by using GPS/BDS dual-mode software receiver.

This paper is structured as follows: Section 2 shows the characteristics of the GPS L1 and BDS B1 signals, and then presents the INS-aided acquisition methodology. In Section 3, the frequency shift and code phase estimation theory are described. In Section 4, the Doppler shift estimation error and the code phase estimation error caused by INS velocity error and position error are derived in detail. Furthermore, the acquisition experiments assisted with different grade INS under high dynamics are performed in Section 5. In Section 6, the final conclusions are given.

## 2. The Principle of INS-Aided Acquisition

### 2.1. Characteristics of GPS L1 Frequency and BDS B1 Frequency Signals Modulation

The C/A and P(Y) signals in the GPS L1 frequency can be written as [2]:

(1) $$S_{L1}(t)=A_{C}C(t)D(t)\sin(2\pi f_{L1}t+\phi_{L1})+A_{P}Y(t)D(t)\cos(2\pi f_{L1}t+\phi_{L1})$$

where $S_{L1}(t)$ is the signal at L1 frequency, $A_{C}$ is the amplitude of the C/A code, C(t) represents the phase of the C/A code, $f_{L1}$ is the L1 frequency, $\phi_{L1}$ is the initial phase, $A_{P}$ is the amplitude of the P(Y) code, Y(t) represents the phase of the P(Y) code, D(t) represents the data code.

The in-phase component (I) and the quadrature component (Q) signals in the BDS B1 frequency are also in quadrant phase of each other and they can be described as follows [18]:

(2) $$S_{B1}(t)=A_{B1I}C_{B1I}(t)D_{B1I}(t)\cos\left(2\pi f_{B1}t+\varphi_{B1I}\right)+A_{B1Q}C_{B1Q}(t)D_{B1Q}(t)\sin\left(2\pi f_{B1}t+\varphi_{B1Q}\right)$$

where $S_{B1}(t)$ is the signal at B1 frequency, $A_{B1I}$ is the amplitude of the I component, $C_{B1I}(t)$ represents the phase of the I component, $f_{B1}$ is the B1 frequency, $\varphi_{B1I}$ is the initial phase, $A_{B1Q}$ is the amplitude of the Q component,$C_{B1Q}(t)$ represents the phase of the Q component, $D_{B1Q}(t)$ represents the data code.

The BDS B1 frequency signal and the GPS L1 frequency signal have many similarities in the center frequency, signal modulation and signal characteristics. A comparison of the characteristics of the BDS B1 frequency signal and GPS L1 frequency signal is shown in Table 1, so the INS–aided acquisition can be verified in GPS/BDS dual-mode software receiver.

### 2.2. INS-Aided Acquisition Methodology

As shown in Figure 1, the GPS and BDS signal acquisition process is a three-dimensional search, including pseudo-random noise code (PRN), code phase and Doppler shift. The GPS satellites are differentiated by 32 different PRN sequences, while the BDS satellites are differentiated by 35 (the number before 2020 is 14) different PRN sequences [1]. From the code phase search direction, the C/A code in GPS L1 frequency contains 1023 chips and the BDS B1I has 2046 chips, however the Doppler shift range is the possible maximum frequency shift. Take the BDS B1I signal for example, there exist 4092 code bins and 21 frequency bins, assuming that the bin width of the code phase search is 1/2 chip, the step size of the Doppler search is 500 Hz and the Doppler span is ±5 kHz. Thus this results in a total of 85,932 search cells, so it is necessary to reduce the range in order to achieve faster acquisition.

The GNSS signal acquisition can be successfully achieved by exceeding the correlation threshold between the local signal and the received signal. Taking the BDS B1I signal for example, the integration output ${\bar{I}}_{P}(n)$ and ${\bar{Q}}_{P}(n)$ are described as follows:

(3) $${\bar{I}}_{P}(n)=AD(n)R\left(\Delta \tau \right)\mathrm{sinc}\left(\pi \Delta fT_{coh}\right)\cos\left(\pi \Delta fT_{coh}+\delta \varphi_{0}\right)+{\bar{N}}_{I}$$

(4) $${\bar{Q}}_{P}(n)=AD(n)R\left(\Delta \tau \right)\mathrm{sinc}\left(\pi \Delta fT_{coh}\right)\sin\left(\pi \Delta fT_{coh}+\delta \varphi_{0}\right)+{\bar{N}}_{Q}$$

where A is the signal amplitude; D(n) is the navigation data; R(Δτ) is the I autocorrelation function; Δτ is the code phase error between received signal and local signal; Δf is the frequency difference between the received signal and local signal; $\delta \phi_{0}$ is the carrier phase error; $T_{coh}$ is the coherent integration time; ${\bar{N}}_{I}$ and ${\bar{N}}_{Q}$ are the white noise.

From Equations (3) and (4), the correlation output is $z_{I}(n)={\bar{I}}_{P}(n)+j\cdot {\bar{Q}}_{P}(n)$. The magnitude of $z_{I}(n)$ can be written as:

(5) $$V_{i}=\sqrt{I_{i}^{2}(n)+Q_{i}^{2}(n)}=AR\left(\Delta \tau \right)\left|\mathrm{sinc}\left(\Delta fT_{coh}\right)\right|+N_{R}$$

**Figure 2:**
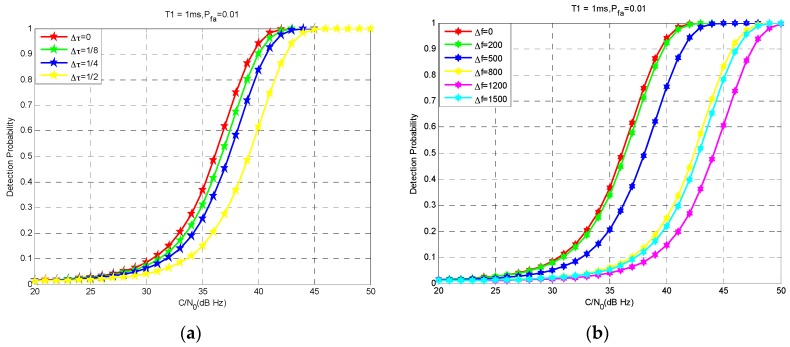
The detection probability with different Δτ and Δf: (**a**) the Δτ is 0, 1/8, 1/4, 1/2 chip; (**b**) the Δf is 0, 200, 500, 800, 1200, 1500 Hz.

Equation (5) shows that the smaller are the code phase error Δτ and the frequency shift Δf, the higher is the detection probability. Under the conditions of $T_{coh}$ = 1 ms and false alarm rate $P_{fa}$ = 0.01, Figure 2a,b show the detection probability when the Δτ is 0, 1/8, 1/4, 1/2 chip and the Δf is 0, 200, 500, 800, 1200 and 1500 Hz, respectively.

From Figure 2, in the same ${C/N}_{0}$, the detection probability is gradually reduced with the increase of the local carrier frequency estimation error and the code phase error. Considering that it is difficult to improve ${C/N}_{0}$, an appropriate means of ensuring the detection probability is by reducing the error of local carrier frequency estimation and code phase estimation. In the INS-aided acquisition, according to the velocity, position information provided by INS and ephemeris, the Doppler frequency shift and code phase can be calculated. The estimated carrier frequency and code phase offset are set as the center of the search range, and the search boundary is set according to the uncertainty of INS information, so this can greatly reduce the carrier frequency and code phase search range. At the same time, the INS-aided acquisition can reduce the error of local carrier frequency estimation and code phase estimation to enhance the detection probability.

**Figure 3:**
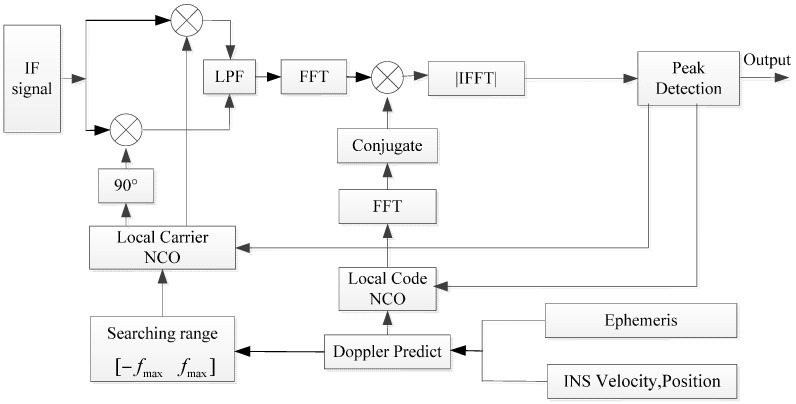
INS-aided acquisition scheme.

As for the search algorithm in the INS-aided acquisition, we choose the software receiver parallel code phase search method [19,20]. The structure of the INS-aided acquisition is shown in Figure 3.

## 3. Frequency Shift and Code Phase Estimation Theory

### 3.1. Frequency Shift Estimation

In the spread of GNSS signal from satellite to receiver, the main factors of Doppler frequency shift are the motion of the receiver relative to the satellite, the receiver clock frequency drift and the satellite clock frequency drift. Therefore, the carrier Doppler frequency shift can be expressed as:

(6) $$f_{d}\;=\;f_{r}-f_{s}+f_{r,c}-f_{s,c}$$

where $f_{r}$ is the Doppler shift on the carrier due to the motion of the receiver with respect to the satellite and $f_{s}$ is the Doppler shift on the carrier due to the motion of the satellite with respect to the receiver. $f_{r,c}$, $f_{s,c}$ are the Doppler frequency shift of the receiver clock frequency drift and satellite clock frequency drift, respectively.

Due to the fact that the satellite uses the atomic clock which has high stability and high precision as time reference and the receiver clock frequency offset can be calibrated locally, the clock error of the satellite $f_{s,c}$ and receiver $f_{r,c}$ can be ignored, so the estimation of Doppler frequency shift in LOS direction can be expressed as:

(7) $$f_{d}=E\;\cdot \;(V_{r}-\;V_{s})/\lambda_{carrier}\;=\;f_{r}-f_{s}$$

where E is the unit vector of the (LOS) and it can be calculated by the relative position of satellite and carrier, $V_{r}$ is the velocity of the receiver, $V_{s}$ is the velocity of the satellite, $\lambda_{carrier}$ is the carrier wavelength and it can be calculated by $\lambda_{carrier}\;=\;c/f_{carrier}$, c is the velocity of light and $f_{carrier}$ is the carrier frequency.

Taking GPS L1 frequency as an example, the maximum radial velocity component of relative motion between the stationary receiver and satellite is about 929 m/s, leading to the maximum Doppler frequency shift is:

(8) $$f_{\mathrm{dM}}\;=\;V_{\mathrm{dM}}\frac{f_{L1}}{c}\;\approx \;\frac{929\times 1575.2\times 10^{6}}{3\times 10^{8}}\;\approx \;4878.8\;\mathrm{Hz}$$

As for the Doppler frequency shift of BDS, it is more complex than GPS. On the one hand, the carrier frequency of BDS is different from GPS. On the other hand, the BDS space constellation includes three kinds of satellites: GEO, IGSO, MEO, and different types of satellites lead to the different maximum Doppler frequency shift. According to the orbit parameters of BDS broadcast, the maximum satellite operation speed can be calculated. The maximum radial velocities of GEO, IGSO and MEO satellite are 13.6 m/s, 424.2 m/s and 864.1 m/s respectively. The maximum Doppler frequency can be calculated by [1]:

(9) $$f_{\mathrm{dM},\mathrm{GEO}}=V_{\mathrm{dM},\mathrm{GEO}}\frac{f_{B1}}{c}\approx \frac{13.6\times 1561.098\times 10^{6}}{3\times 10^{8}}\approx 70.8\;\mathrm{Hz}$$

(10) $$f_{\mathrm{dM},\mathrm{IGSO}}=V_{\mathrm{dM},\mathrm{IGSO}}\frac{f_{B1}}{c}\approx \frac{424.2\times 1561.098\times 10^{6}}{3\times 10^{8}}\approx 2207.4\;\mathrm{Hz}$$

(11) $$f_{\mathrm{dM},\mathrm{MEO}}=V_{\mathrm{dM},\mathrm{MEO}}\frac{f_{B1}}{c}\approx \frac{864.1\times 1561.098\times 10^{6}}{3\times 10^{8}}\approx 4496.5\;\mathrm{Hz}$$

From the above analysis, the maximum Doppler shift caused by the satellite motion is about 5 kHz for a stationary receiver in the earth’s surface. As shown in Equation (7), the Doppler shift caused by the receiver motion can be calculated by the velocity and position of INS.

### 3.2. Code Phase Estimation

Taking GPS as an example, the first PRN chip and the first bit of the 50 bits per second (bps) data stream are sent out from every satellite at the midnight of Saturday in GPS time, so the code phase can be calculated according to the signal sending time of GPS, and expressed as follows:

(12) $$\tau \;=\;\mathrm{mod}\;(1023\;\times \;t_{\mathrm{SV}}\times \;1000,1023)$$

where $t_{\mathrm{SV}}$ is the signal sending time of GPS, which can be calculated using the signal receiving time $t_{R}$, the signal transmitting time $D_{t}$ and the clock error $t_{c}$. Therefore the signal sending time estimation ${\hat{t}}_{\mathrm{SV}}$ is described as:

(13) $${\hat{t}}_{\mathrm{SV}}\;=\;t_{R}-D_{t}+t_{c}$$

The code phase estimation of the received signal $\hat{\tau }$ is:

(14) $$\hat{\tau }\;=\;\mathrm{mod}[1023\;\times \;(t_{R}-D_{t}+t_{c})\;\times \;1000,1023]$$

where $t_{c}$ is provided by GNSS ephemeris, $D_{t}=E\cdot (P_{r}-P_{s})/c$, $P_{r}$ and $P_{s}$ represent the carrier position and the satellite position. The code phase estimation can be calculated by INS position which error is dependent on the INS accuracy.

## 4. Doppler Shift Estimation Error and Code Phase Estimation Error

**Figure 4:**
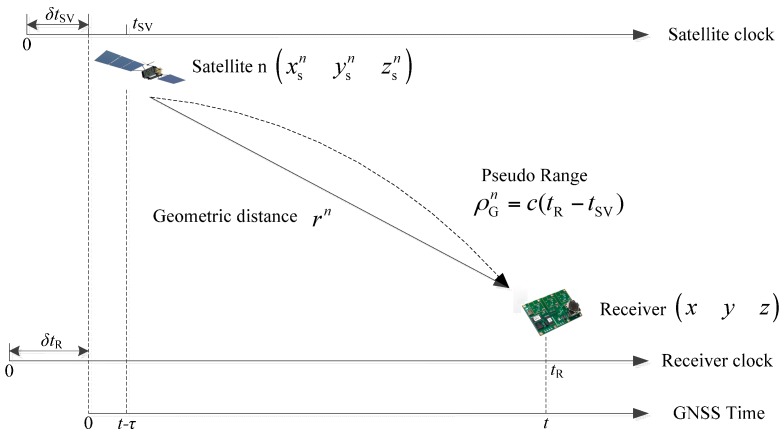
Satellite pseudo-range scheme.

As shown in Figure 4, assuming the position of the satellite n is $\left(\begin{array}{ccc} x_{s}^{n} & y_{s}^{n} & z_{s}^{n} \end{array}\right)$, the receiver‘s real position is $\left(\begin{array}{ccc} x & y & z \end{array}\right)$ and the position of INS is $\left(\begin{array}{ccc} x_{I} & y_{I} & z_{I} \end{array}\right)$. So the pseudo-range $\rho_{G}^{n}$ from the receiver to the satellite and the pseudo-range $\rho_{I}^{n}$ from the INS to the satellite can be expressed as follows:

(15) $$\rho_{G}^{n}=c(t_{R}-t_{\mathrm{SV}})=r^{n}+\text{​}c\delta t+n_{\rho }$$

(16) $$\rho_{I}^{n}=\sqrt{{\left(x_{I}-x_{s}^{n}\right)}^{2}+{\left(y_{I}-y_{s}^{n}\right)}^{2}+{\left(z_{I}-z_{s}^{n}\right)}^{2}}$$

where δt is the clock error, $r^{n}=\sqrt{{\left(x-x_{s}^{n}\right)}^{2}+{\left(y-y_{s}^{n}\right)}^{2}+{\left(z-z_{s}^{n}\right)}^{2}}$ is the geometric distance from the satellite to the receiver, $n_{\rho }$ is the error of noise.

Equation (16) is executed in Taylor expansion, and the following expression is obtained:

(17) $$\rho_{I}^{n}\approx \sqrt{{\left(x-x_{s}^{n}\right)}^{2}+{\left(y-y_{s}^{n}\right)}^{2}+{\left(z-z_{s}^{n}\right)}^{2}}\;+\;\frac{\partial \rho_{I}^{n}}{\partial x}(x_{I}-x)\;+\;\frac{\partial \rho_{I}^{n}}{\partial y}(y_{I}-y)\;+\;\frac{\partial \rho_{I}^{n}}{\partial z}(z_{I}-z)$$

Assuming $\delta x\;=\;x_{I}\;-\;x$, $\delta y\;=\;y_{I}\;-\;y$, $\delta z\;=\;z_{I}-\;z$, Equation (17) can be expressed as:

(18) $$\rho_{I}^{n}=\;r^{n}\;+\;\frac{\partial \rho_{I}^{n}}{\partial x}\delta x\;+\;\frac{\partial \rho_{I}^{n}}{\partial y}\delta y\;+\;\frac{\partial \rho_{I}^{n}}{\partial z}\delta z$$

(19) $$\left\{ \begin{array}{l} \frac{\partial \rho_{I}^{n}}{\partial x_{u}}=\frac{x-x_{s}^{n}}{\sqrt{{\left(x-x_{s}^{n}\right)}^{2}+{\left(y-y_{s}^{n}\right)}^{2}+{\left(z-z_{s}^{n}\right)}^{2}}}=\frac{x-x_{s}^{n}}{r^{n}}=e_{1}\\ \frac{\partial \rho_{I}^{n}}{\partial y_{u}}=\frac{y-y_{s}^{n}}{\sqrt{{\left(x-x_{s}^{n}\right)}^{2}+{\left(y-y_{s}^{n}\right)}^{2}+{\left(z-z_{s}^{n}\right)}^{2}}}=\frac{y-y_{s}^{n}}{r^{n}}=e_{2}\\ \frac{\partial \rho_{I}^{n}}{\partial z_{u}}=\frac{z-z_{s}^{n}}{\sqrt{{\left(x-x_{s}^{n}\right)}^{2}+{\left(y-y_{s}^{n}\right)}^{2}+{\left(z-z_{s}^{n}\right)}^{2}}}=\frac{z-z_{s}^{n}}{r^{n}}=e_{3} \end{array}\right.$$

where $E={\left[\begin{array}{ccc} e_{1} & e_{2} & e_{3} \end{array}\right]}^{T}$ is the unit vector of the LOS.

The pseudo-range $\rho_{I}^{n}$ from the INS to the satellite is:

(20) $$\rho_{I}^{n}=r^{n}\;+\;e_{1}\delta x\;+\;e_{2}\delta y\;+\;e_{3}\delta z$$

The pseudo-range rate ${\dot{\rho }}_{I}^{n}$ from the INS to the satellite can be described by:

(21) $$\begin{array}{cc} {\dot{\rho }}_{I}^{n} & =e_{1}({\dot{x}}_{I}-{\dot{x}}_{s}^{n})\;+\;e_{2}({\dot{y}}_{I}-{\dot{y}}_{s}^{n})\;+\;e_{3}({\dot{z}}_{I}-{\dot{z}}_{s}^{n})\\ & =e_{1}(\dot{x}-{\dot{x}}_{s}^{n})\;+\;e_{2}(\dot{y}-{\dot{y}}_{s}^{n})\;+\;e_{3}(\dot{z}-{\dot{z}}_{s}^{n})\;+\;e_{1}\delta \dot{x}\;+\;e_{2}\delta \dot{y}\;+\;e_{3}\delta \dot{z} \end{array}$$

The pseudo-range rate ${\dot{\rho }}_{G}^{n}$ from the receiver to the satellite can be described by:

(22) $${\dot{\rho }}_{G}^{n}=e_{1}(\dot{x}-{\dot{x}}_{s}^{n})\;+\;e_{2}(\dot{y}-{\dot{y}}_{s}^{n})\;+\;e_{3}(\dot{z}-{\dot{z}}_{s}^{n})+\text{​}c\delta \dot{t}+{\dot{n}}_{\rho }$$

From Equations (15) and (20)–(22), the pseudo-range error $\delta \rho^{n}$ and pseudo-range rate error $\delta {\dot{\rho }}^{n}$ can be described by:

(23) $$\delta \rho^{n}=\rho_{I}^{n}-\rho_{G}^{n}=e_{1}\delta x\;+\;e_{2}\delta y\;+\;e_{3}\delta z-c\delta t-n_{\rho }$$

(24) $$\delta {\dot{\rho }}^{n}={\dot{\rho }}_{I}^{n}-{\dot{\rho }}_{G}^{n}=e_{1}\delta \dot{x}\;+\;e_{2}\delta \dot{y}\;+\;e_{3}\delta \dot{z}-c\delta \dot{t}-{\dot{n}}_{\rho }$$

where δx,δy,δz are the position error, and $\delta \dot{x},\delta \dot{y},\delta \dot{z}$ are the velocity errors of INS in the space rectangular coordinate system. In the geodetic coordinate system, they can be expressed using the following equations:

(25) $$\left\{ \begin{array}{l} \delta x\;=\;\delta h\cos L\cos\lambda -(R_{N}+h)\sin L\cos\lambda \delta L-\;(R_{N}+h)\cos L\sin\lambda \delta \lambda \\ \delta y\;=\;\delta h\cos L\sin\lambda -(R_{N}+h)\sin L\sin\lambda \delta L+\;(R_{N}+h)\cos L\cos\lambda \delta \lambda \\ \delta z\;=\;\delta h\sin L+[R_{N}(1-e^{2})+h]\cos L\delta L \end{array}\right.$$

(26) $$\left\{ \begin{array}{l} \delta \dot{x}\;=-\delta v_{E}\sin\lambda -\delta v_{N}\sin L\cos\lambda +\delta v_{U}\cos L\cos\lambda \\ \delta \dot{y}\;=\;\delta v_{E}\cos\lambda -\delta v_{N}\sin L\sin\lambda +\delta v_{U}\cos L\sin\lambda \\ \delta \dot{z}\;=\;\delta v_{N}\cos L+\delta v_{U}\sin L \end{array}\right.$$

where L,λ,h represent latitude, longitude and altitude, respectively. δL, δλ, δh are the latitude error, longitude error and altitude error, respectively. ${\left[\begin{array}{ccc} \delta v_{E} & \delta v_{N} & \delta v_{U} \end{array}\right]}^{T}$ is the vector of the receiver velocity error in the navigation frame.

The code phase estimation error $\Delta \tau_{I}$ and the Doppler estimation error $\Delta f_{I}$ caused by INS velocity error and position error have the following relations with $\delta \rho^{n}$ and $\delta {\dot{\rho }}^{n}$:

(27) $$\lambda_{\mathrm{code}}\Delta \tau_{I}\;=\;\delta \rho \;=\;e_{1}\delta x\;+\;e_{2}\delta y\;+\;e_{3}\delta z-c\delta t-n_{\rho }$$

(28) $$\lambda_{\mathrm{carrier}}\Delta f_{I}\;=\;\delta \dot{\rho }\;=\;e_{1}\delta \dot{x}\;+\;e_{2}\delta \dot{y}\;+\;e_{3}\delta \dot{z}-c\delta \dot{t}-{\dot{n}}_{\rho }$$

where $\lambda_{\mathrm{code}}$ is the code phase wavelength and $\lambda_{carrier}$ is the carrier wavelength, δt and $\delta \dot{t}$ are the clock error and clock shift which can be calibrated locally.

INS velocity and position error are mainly determined by the bias and shifts of gyros and accelerometers. The dynamic equations for a strapdown INS are given by:

(29) $$\delta {\dot{v}}^{n}=-\left(\delta \omega_{\mathrm{en}}^{n}+2\delta \omega_{\mathrm{ie}}^{n}\right)\;\times \;v^{n}-\left(\omega_{\mathrm{en}}^{n}+2\omega_{\mathrm{ie}}^{n}\right)\;\times \;\delta v^{n}+C_{b}^{n}f^{b}\times \;\varphi +C_{b}^{n}\nabla^{b}$$

(30) $$\left\{ \begin{array}{l} \delta \dot{L}=\frac{1}{R_{M}+h}\delta v_{N}\\ \delta \dot{\lambda }=\frac{\sec L}{R_{N}+h}\delta v_{E}+\frac{v_{E}}{R_{N}+h}\sec L\tan L\delta L\\ \delta \dot{h}=\delta v_{U} \end{array}\right.$$

where $R_{M}$ is the transverse curvature radii and $R_{N}$ is the meridian curvature radii. $\omega_{\mathrm{ie}}^{n}$ is the Earth’s rotation rate in the navigation frame. $\omega_{\mathrm{en}}^{n}$ is the angular rate of the navigation frame with respect to the Earth frame. $\delta \omega_{\mathrm{ie}}^{n}$ is the error of the Earth rotation rate. $v^{n}={\left[v_{E},v_{N},v_{U}\right]}^{T}$ is the velocity vector in the navigation frame coordinates. $f^{b}$ is the accelerometer’s output specific force vector in the body frame, φ is the attitude error vector of the body frame with respect to the navigation frame. $C_{b}^{n}$ is the transformation matrix from the computed body frame to the navigation frame. $\nabla^{b}$ is the accelerometer error vector in the body frame. Combined Equations (25)–(30), $\Delta \tau_{I}$ and $\Delta f_{I}$ can be estimated by the INS velocity error and position error which are relevant to the precision of gyros and accelerometers.

## 5. The INS-Aided Acquisition Experiments with Different Grade INS

**Figure 5:**
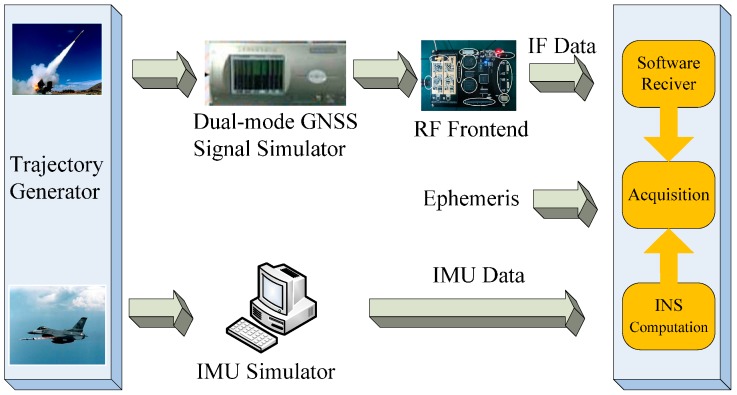
The block diagram of simulation platform.

**Figure 6:**
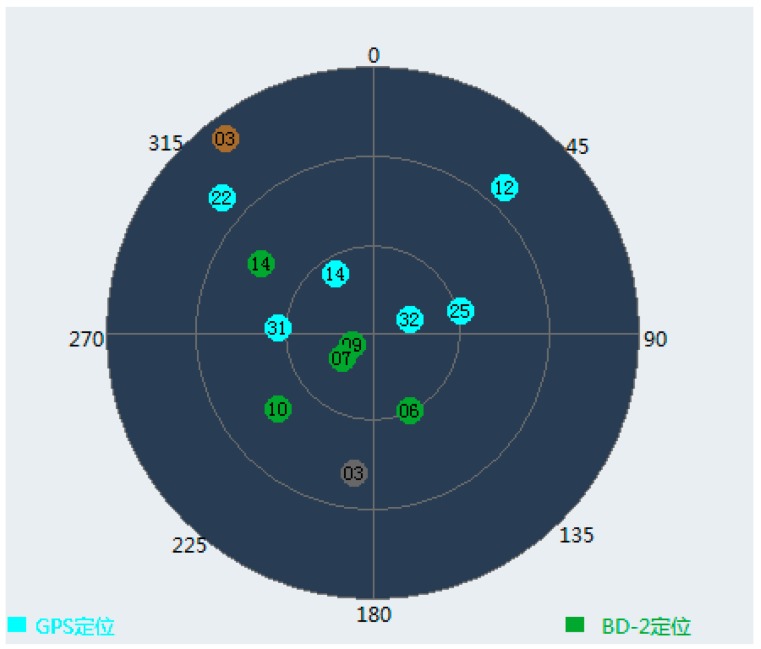
The constellation of BDS and GPS in the simulation.

**Figure 7:**
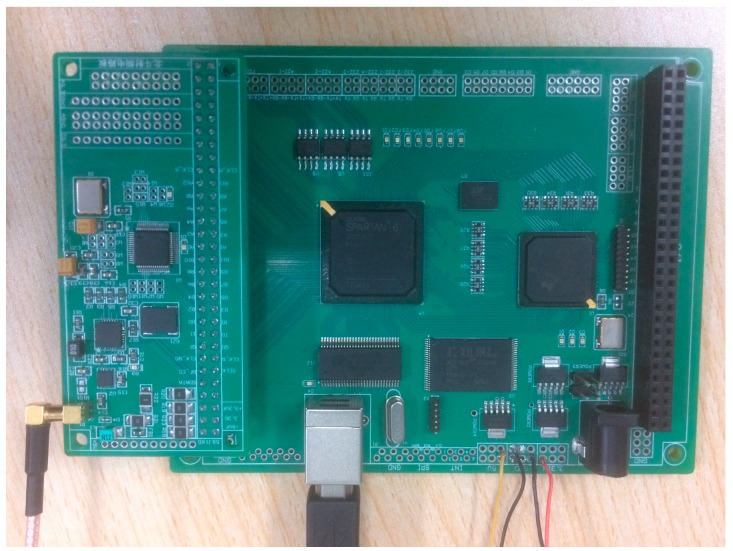
The RF frontend designed based on a MAX2769.

In order to analyze the performance of different grade INS in the INS-aided acquisition experiments, a software simulation platform was firstly designed. As shown in Figure 5, the block diagram of the simulation platform includes: trajectory generator module, dual-mode GNSS signal simulation module, INS generating module and GNSS software receiver module [21]. Then, a trajectory with high dynamics, which is similar with missiles and aircrafts trajectory, is designed and simulated by the trajectory generator module. Under these conditions, the constellation of BDS and GPS are built. As shown in Figure 6, the constellation of BDS and GPS each has six satellites respectively in the simulation. Then, a dual-mode GNSS signal simulator is used to transmit the satellite radio frequency (RF) signal, which is nearly the same as the real signal. After that, the RF signal is received by an RF frontend which is designed based on MAX2769, as shown in Figure 7. Later, the received RF signal is converted to the intermediate frequency (IF) digital in the process of software reception. The gyro and acceleration data of different grade INS are simulated using the designed trajectory. Finally, the IF data and IMU data are input into the GNSS software receiver module for INS-aided acquisition experiments.

### 5.1. Simulation Scenario Design

**Figure 8:**
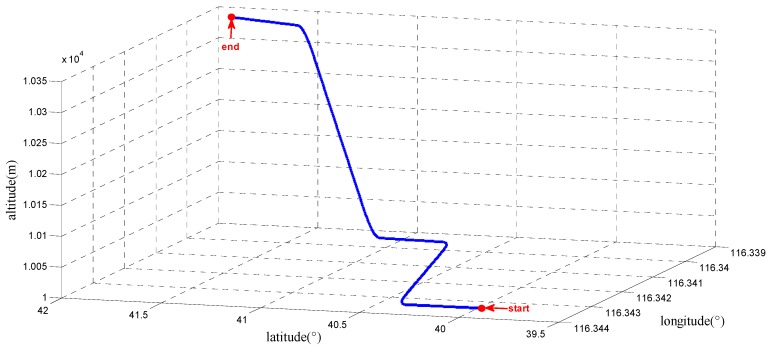
The three-dimensional high dynamic trajectory in the geodetic frame.

In order to confirm the performance of the INS-aided acquisition under highly dynamic conditions, a highly dynamic simulation scenario is designed. The trajectory includes acceleration, uniform motion, turning and climbing. The trajectory parameters, which include the acceleration and angular rate in the body frame, are listed in Table 2. The initial latitude, longitude and altitude of the trajectory is [39.977886°, 116.343400°, 10,000 m], and the initial velocity is [0 m/s, 200 m/s, 0 m/s]. The highly dynamic trajectory according to the designed scenario is depicted in Figure 8.

### 5.2. Parameters of Different Grade INS

The INS plays an important role in the progress of signal acquisition and tracking. Considering that the INS can offer the relative position and velocity of the receiver with respect to the satellite, which are the core parameters used to calculate the Doppler shift and the code phase, in the INS-aided acquisition experiments, we use the gyro and accelerometer data generator to calculate the INS information and the generator is developed based on the mathematical simulation model given in [22].

In the gyro and accelerometer data generator, the true angular rate and acceleration are listed in Table 2. Then, in order to simulate the outputs, the gyro and accelerometer errors which include constant biases, random drifts and noises are added into the true value. The gyro and accelerometer errors can lead to the reflecting constant bias, bias stability and random walk.

In Equation (29), different grades of INS bring different velocity and position errors. This would then affect the acquisition results due to the different Doppler shift and code phase ranges. Thus, for the purpose to analysis the performance changes caused by different INS grades, a variety of INS are simulated in the designed scenario. The parameters of the selected INS in the experiments are listed in Table 3. In the above analysis, the precision of INS varies by 10 times, which covers the typical precision used in the current application.

### 5.3. Velocity and Position Error Using Different INS

**Figure 9:**
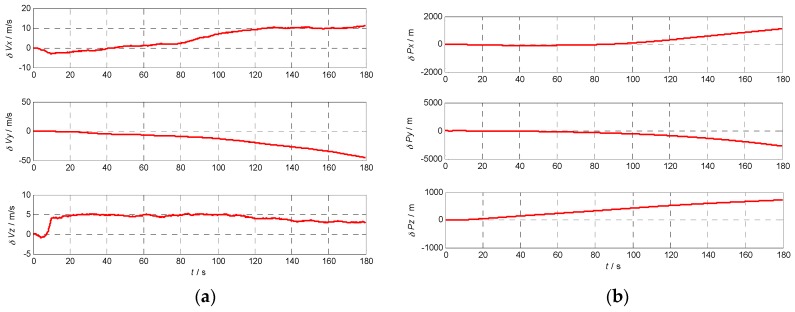
Velocity and position errors of MEMS grade INS: (**a**) the velocity error curve of MEMS grade INS; (**b**) the position error curve of MEMS grade INS.

**Figure 10:**
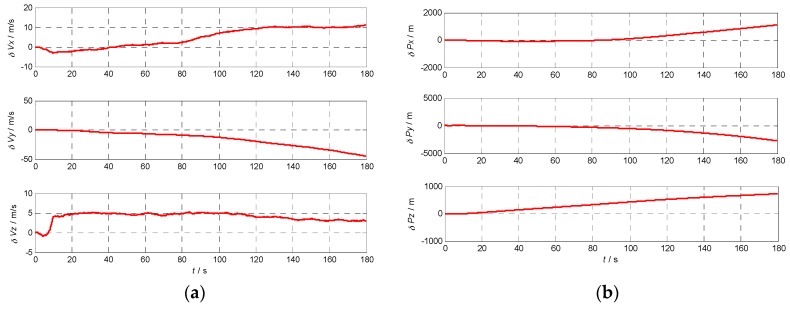
Velocity and position errors of civil grade INS: (**a**) the velocity error curve of civil grade INS; (**b**) the position error curve of civil grade INS.

**Figure 11:**
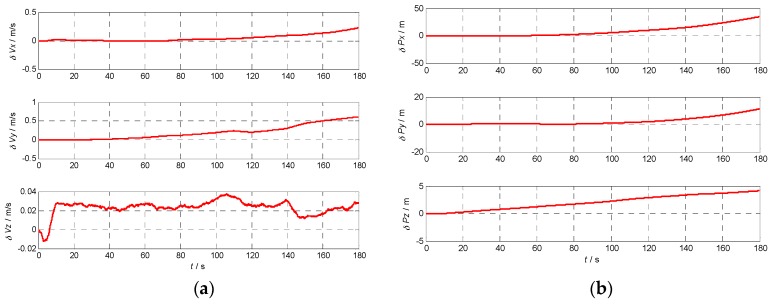
Velocity and position errors of tactical grade INS: (**a**) the velocity error curve of tactical grade INS; (**b**) the position error curve of tactical grade INS.

**Figure 12:**
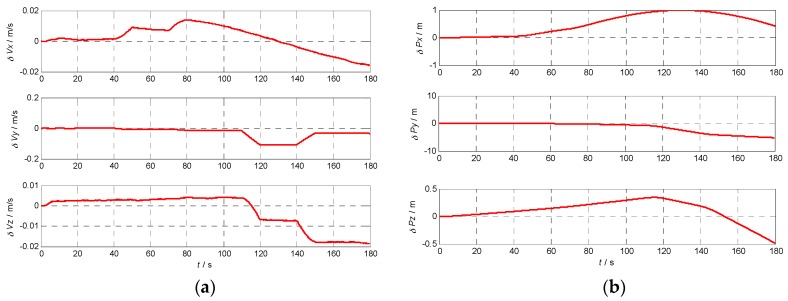
Velocity and position errors of inertial grade INS: (**a**) the velocity error curve of inertial grade INS; (**b**) the position error curve of inertial grade INS.

In order to analyze the effects of the INS errors on the performance of Doppler shift and code phase estimations conveniently, the initial position and velocity are assumed known without errors. To calculate the Doppler shift error and code phase error, the relative position and velocity errors between the receiver and the satellite caused by the gyro and accelerometer errors are illustrated in Figure 9, Figure 10, Figure 11 and Figure 12 with the four different grades of INS, and the maximum errors in 180 s are further listed in Table 4. From the above results, we can draw the conclusion that the velocity and position errors increase as the INS decrease in quality.

### 5.4. Doppler Shift and Code Phase Estimation Errors Using Different Grade INS

According to Equations (27) and (28), Doppler shift and code phase estimation errors exist due to the velocity error and positon error caused by the INS used. According to the ephemeris and the INS information of the 12 visible satellites, the Doppler shift and the code phase estimation of each satellite can be calculated by Equations (7) and (14). Then, the Doppler shift and code phase estimation errors of the 12 visible satellites are achieved by calculating the difference between the estimation and real value. The maximum Doppler shift estimation errors and the maximum code phase estimation errors are listed in Table 5 and Table 6, respectively.

The experimental results show that the Doppler shift and code phase estimation errors increase as the quality of the INS decreases. The Doppler shift estimation errors are less than the minimal frequency search space (±500 Hz) in 180 s. The code phase estimation errors are less than 1023 chips (for GPS) or 2046 chips (for BDS). Furthermore, by comparing Table 3 with Table 5, it can be seen that the Doppler shift estimation error is about 200 Hz when using a MEMS grade INS, and that if the INS accuracy increases by 10-fold, the estimation error of the Doppler frequency shift decreases by 10-fold. Similarly, the code phase estimation error decreases approximately 10-fold if the INS accuracy improves by 10-fold. Thus, the appropriate INS for the intended acquisition can be selected according to the required accuracy of the Doppler frequency shift and code phase.

### 5.5. INS-Aided Acquisition Experiments and Results

Due to the fact that the clock error of the software receiver has been compensated before the INS-aided acquisition experiments and the satellite ephemeris is true, the residual frequency shift is the Doppler shift caused by the INS position and velocity errors. In this case, the performance of the INS-aided acquisition can be fully verified. In Table 7, the search parameters with different grades of INS-aided and no INS-aid acquisition is illustrated. Due to the decrease of frequency and code phase range, the acquisition time of the INS-aided case is shorter than no INS-aid case. Besides, if the frequency search space is smaller, the accuracy of the Doppler shift estimation is higher.

The selected MEMS-grade INS has the same search parameters as the selected higher grade INS. In other words, the selected MEMS-grade INS can satisfy the demands of fast acquisition under highly dynamic conditions. Considering the price, the weight and the size, the selected MEMS-grade INS is the best choice for the deeply coupled integration.

After the signal interruption, the navigation information provided by INS can not only be used to save acquisition time, but also it can keep tracking in a short time. If the LOS error estimated by the local clock and INS position is less than half a chip, instantaneous acquisition can be achieved in the code phase direction. Similarly, the instantaneous acquisition of carrier frequency can be realized if the frequency error estimated by SINS is less than the equivalent bandwidth of the tracking loop. This offers great advantages in applications when the signal is frequently interrupted but the duration of the interruption is short, such as urban vehicle navigation.

## 6. Conclusions

GNSS receivers cannot work and frequently lose lock under highly dynamic conditions due to the large Doppler frequency shift, which causes difficulties for the fast acquisition and re-acquisition of signals. The INS-aided acquisition can improve the acquisition performance by estimating and compensating the Doppler frequency shift and the code phase. This paper analyzes in depth the performance of GNSS signal acquisition aided by different grades of INS under highly dynamic conditions. In order to estimate and compensate the Doppler frequency shifts as well as the code phase, the error sources of the Doppler shift and code phase estimation are analyzed. Furthermore, the mathematical model of Doppler shift and code phase estimation errors contributed by the INS velocity error and position error are derived in detail. In order to analyze the effects of the INS quality on the Doppler shift and code phase estimation accuracy, experiments are performed using different grades of INS under a highly dynamic scenario. Then, the INS-aided acquisition is simulated in a dual-mode software receiver. The simulation results show that the acquisition aided by INS can reduce the frequency and code phase search space under highly dynamic conditions. Moreover, the Doppler shift and code phase estimation errors increase as the quality of the INS decreases. The quantitative result is that the acquisition time can be shortened by about 1.85 s with the assistance of the selected INS. Compared with a high grade INS, the selected MEMS-grade INS provides the same improvement. It can be seen that the selected MEMS-grade INS can satisfy the demands of fast acquisition in the simulation scenario, and according to the theoretical analysis, experiments and simulations, a suitable INS can be chosen for the deeply coupled integration.

## Figures and Tables

**Table 1:** Comparison of the characteristics of the BDS B1 frequency signal and the GPS L1 frequency signal.

Parameter	BDS B1 Signal	GPS L1 Signal
Center Freq. (MHz)	1561.098 MHz	1575.42 MHz
Modulation Type	QPSK-CDMA	BPSK-CDMA
Code Rate (MHz)	2.046	1.023
Code Length (chip)	2046	1023
Symbol Rate	GEO 500 bps, MEO/IGSO 50 bps	50 bps

**Table 2:** The trajectory parameters of simulation in high dynamic applications.

Motion State	Time (s)	Angular Rate (°/s)			Acceleration (m/s^2^)		
		*x* Axis	*y* Axis	*z* Axis	*x* Axis	*y* Axis	*z* Axis
Acceleration	10	0	0	0	0	100	0
Uniform motion	30	0	0	0	0	0	0
Turning right	10	0	0	9	−1.57	0	0
Uniform motion	20	0	0	0	0	0	0
Turning left	10	0	0	−9	1.57	0	0
Uniform motion	30	0	0	0	0	0	0
Climbing	10	10	0	0	0	0	1.74
Uniform motion	20	0	0	0	0	0	0
Yielding	10	−10	0	0	0	0	−1.74
Uniform motion	30	0	0	0	0	0	0

**Table 3:** The parameters of different grade INS.

IMU Parameters		MEMS Grade	Civil Grade	Tactical Grade	Inertial Grade
Gyro	Constant bias (°/h)	10	1	0.1	0.01
	Bias stability (°/h)	10	1	0.1	0.01
	Random walk (°/$\sqrt{h}$)	1	0.1	0.01	0.001
Accelerometer	Constant bias (mg)	10	1	0.1	0.01
	Bias stability (mg)	10	1	0.1	0.01
	Random walk (mg/$\sqrt{\mathrm{Hz}}$)	1	0.1	0.01	0.001

**Table 4:** Maximum position and velocity errors of the different grade INS.

Maximum Error		MEMS Grade	Civil Grade	Tactical Grade	Inertial Grade
Velocity (m/s)	*x* axis	11.14	1.88	0.22	−0.016
	*y* axis	−45.09	−4.98	0.60	−0.1085
	*z* axis	5.19	0.52	0.04	−0.0183
Position (m)	*x* axis	1115	265.5	11.33	0.99
	*y* axis	−2737	−328.6	34.66	−5.23
	*z* axis	725.2	73.2	4.18	−0.48

**Table 5:** Maximum Doppler shift estimation errors of total satellites.

		MEMS Grade	Civil Grade	Tactical Grade	Inertial Grade
BDS	PRN 3	164.0205	19.8074	2.2807	0.3910
	PRN 6	112.2213	13.1841	1.4684	0.2656
	PRN 7	139.3393	16.5499	1.8787	0.3185
	PRN 9	134.6166	15.6793	1.7725	0.3139
	PRN 10	161.1931	18.9910	2.1786	0.3649
	PRN 14	190.0555	22.9295	2.6264	0.4121
GPS	PRN 12	188.4075	22.3989	2.6002	0.4160
	PRN 14	79.7551	8.8936	0.9501	0.2053
	PRN 22	85.3412	9.8542	1.0636	0.2127
	PRN 25	196.1639	22.2265	2.5813	0.4490
	PRN 31	218.4609	25.5183	3.0269	0.4730
	PRN 32	129.8616	16.5743	1.8918	0.2780

**Table 6:** Maximum code phase estimation errors of total satellites.

		MEMS Grade	Civil Grade	Tactical Grade	Inertial Grade
BDS	PRN 3	16.6109	2.3727	0.1767	0.0229
	PRN 6	12.3420	1.6628	0.1198	0.0154
	PRN 7	14.5754	2.0277	0.1488	0.0192
	PRN 9	14.0123	1.8668	0.1423	0.0186
	PRN 10	16.1468	2.2217	0.1707	0.0224
	PRN 14	18.3990	2.6928	0.2032	0.0264
GPS	PRN 12	9.0416	1.2784	0.0988	0.0130
	PRN 14	4.6490	0.5560	0.0408	0.0054
	PRN 22	5.0048	0.6440	0.0445	0.0057
	PRN 25	8.9826	1.1339	0.0998	0.0138
	PRN 31	9.4901	1.3072	0.1128	0.0154
	PRN 32	7.0746	1.1309	0.0714	0.0086

**Table 7:** Search parameters with different grades of INS.

	No INS Assisted	MEMS Grade	Civil Grade	Tactical Grade	Inertial Grade
Number of satellites	12	12	12	12	12
Frequency range	±5 KHz	±500 Hz	±500 Hz	±500 Hz	±500 Hz
Frequency search space	500 Hz	500 Hz	500 Hz	500 Hz	500 Hz
Code phase range	GPS:1023	GPS:10	GPS:10	GPS:10	GPS:10
	BDS:2046	BDS:20	BDS:20	BDS:20	BDS:20
Code phase search space	0.5	0.5	0.5	0.5	0.5
Search cells	GPS: 42966	GPS:60	GPS:60	GPS:60	GPS:60
	BDS: 85932	BDS:120	BDS:120	BDS:120	BDS:120
Acquisition time	12.87s	1.85s	1.85s	1.85s	1.85s
